# Partial agonist antipsychotic drugs differentially interact with a secondary binding site at the dopamine D_2_ receptor

**DOI:** 10.1093/ijnp/pyaf076

**Published:** 2025-10-29

**Authors:** Richard Ågren, Kristoffer Sahlholm

**Affiliations:** Department of Physiology and Pharmacology, Karolinska Institutet, Stockholm, Sweden; Department of Neurosurgery, Karolinska University Hospital, Stockholm, Sweden; Department of Physiology and Pharmacology, Karolinska Institutet, Stockholm, Sweden; Department of Medical and Translational Biology, Umeå University, Umeå, Sweden; Wallenberg Centre for Molecular Medicine, Umeå University, Umeå, Sweden

**Keywords:** dopamine D_2_ receptor, antipsychotic drugs, secondary binding pocket, bivalent ligand

## Abstract

**Graphical Abstract:**
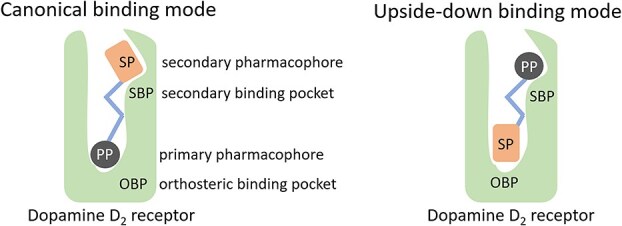

Significance Statement

- Aripiprazole and brexpiprazole showed significantly increased dissociation from the dopamine D2 receptor upon L94A mutation, indicating that their (similar) secondary pharmacophores interact with the secondary binding pocket.
- Cariprazine, sharing an identical primary pharmacophore with aripiprazole, but with a distinct secondary pharmacophore, was unaffected by the secondary binding pocket mutation. 
- In summary, these data are congruent with a canonical binding pose of phenylpiperazine dopamine D2 partial agonists.
- In contrast to aripiprazole, cariprazine, and brexpiprazole, lumateperone lacked detectable dopamine D2 receptor efficacy in our assay.

Dopamine D_2_ receptor (D_2_R) partial agonist antipsychotics (DPAs) provide an improved side-effect profile compared to D_2_R antagonists. Recent structural and computational data suggest an “upside-down” binding mode, differing from an earlier envisaged “canonical” mode, of these antipsychotics in the D_2_R. We experimentally evaluated all four currently approved DPAs to interrogate their binding poses at the D_2_R. Perturbation of a secondary binding pocket (SBP) substantially influenced the dissociation of aripiprazole and brexpiprazole, but not of cariprazine and lumateperone. Considering structural differences and similarities between the four antipsychotics, these findings support canonical D_2_R binding poses of aripiprazole analogues.

Antipsychotics, the majority of which are D_2_R antagonists, are fundamental for treating psychotic disorders. D_2_R antagonism can provoke extrapyramidal symptoms and hyperprolactinemia, but such side effects are less common with DPAs, including the analogues aripiprazole, cariprazine, and brexpiprazole, which are partial D_2_R agonists.^1^ These drugs contain two pharmacophores; the primary pharmacophore (PP), consisting of a phenylpiperazine moiety and earlier considered to interact with the D_2_R orthosteric binding pocket (OBP), connected to a secondary pharmacophore (SP) by an alkyl linker (Figure 1A). The fourth DPA, lumateperone, was included for completeness.

**Figure 1:**
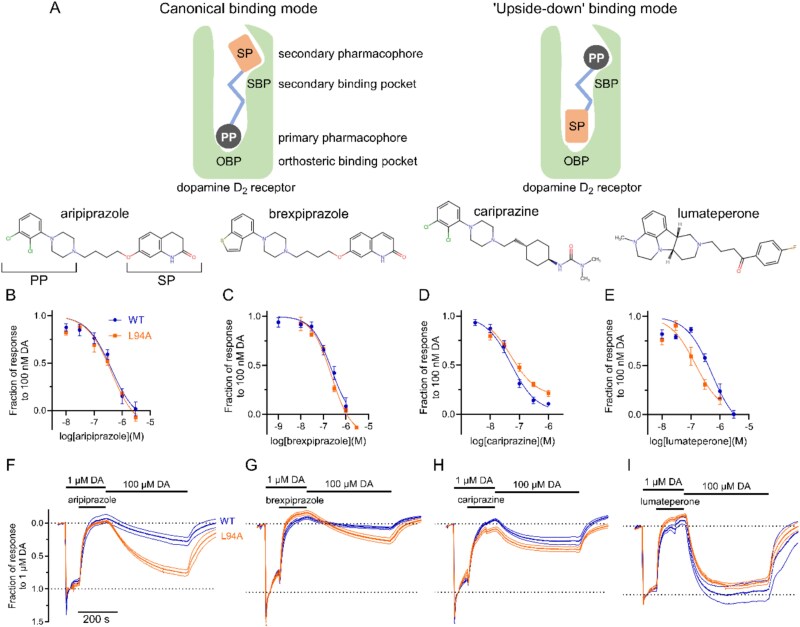
Partial agonist binding to D_2_R WT and L94A. (A) Proposed binding modes and structures of the DPAs. (B-E) Concentration-response relationships of (B) aripiprazole, (C) brexpiprazole, (D) cariprazine, and (E) lumateperone in the presence of 100 nM dopamine (DA). The GIRK currents were normalized to the response evoked by DA alone. Sigmoidal functions were fit to data normalized to the maximum response evoked by DA. (F-I) dopamine-normalized time-averaged dopamine response recovery at D_2_R WT and L94A for 3 μM aripiprazole (F), brexpiprazole (G), cariprazine (H), and 10 μM lumateperone (I).

The SP has been considered to interact with D_2_R extracellular loops 1 and 2 (ECL1-2), which form part of an SBP influencing specificity,^2^ efficacy,^3^ and induced-fit trapping^4^ of ligands. Interactions between L94 and W100 are central for this SBP and L94A mutation has been shown to increase the dissociation of an aripiprazole analogue, SV-III-130.^4^

Recently, crystal structures of the serotonin _2A_ receptor (5-HT_2A_R) bound to aripiprazole and cariprazine revealed unexpected “upside-down” poses, in which the PPs of these antipsychotics were aligned towards the SBP, while docking studies suggested similar binding poses for both ligands also at the D_2_R,^5^ challenging previous views. Given the identical PPs of aripiprazole and cariprazine (Figure 1A), binding differences between these drugs should be dependent on their SPs. Here, we used the L94A mutant to interrogate the likely binding poses of DPAs at the D_2_R.

Oocytes from the African clawed frog, *Xenopus laevis*, were isolated and microinjected with cRNA encoding human long *D_2_R*, *G protein-coupled inward rectifying potassium channel 1 and 4* (*GIRK1/4*), and *regulator of G protein signaling 4* (*RGS4*) as described previously,^6^ using procedures approved by the Animal Welfare Ethical Committee in Stockholm. D_2_R activation-induced GIRK1/4 potassium currents were recorded in high-potassium (25 mM KCl) solution using two-electrode voltage-clamp electrophysiology.^4^

First, aripiprazole, brexpiprazole, cariprazine, and lumateperone inhibitory potencies at WT and L94A D_2_R were evaluated by co-application with 100 nM dopamine (Figure 1B-E). Only lumateperone showed a 5-fold increase in potency at L94A (Table 1). Induced-fit trapping was investigated by application of dopamine to oocytes expressing D_2_R, followed by a partial agonist ligand during 120 s, and finally a supramaximal (100 μM) dopamine concentration to displace the ligand. Whereas L94A mutation increased response recovery from aripiprazole and brexpiprazole by 2.7- and 3.1-fold, respectively (Figure 1F-G), the substitution had no significant effect on cariprazine (Figure 1H). Full response recovery from lumateperone was observed with both WT and L94A mutant D_2_R (Figure 1I). Finally, D_2_R agonist efficacy was evaluated. Despite generally being considered a DPA, lumateperone displayed no intrinsic activity, in contrast to the partial agonism observed with 3 μM of aripiprazole, brexpiprazole, and cariprazine (Table 1).

**Table 1:** DPA potencies, displacement, and partial agonism at WT and L94A mutant D_2_R.

**Ligand**	**D**_**2**_ **R variant**	**Potency** **(pK**_**i**_**, 95% C.I.)**	**Recovery ± SEM** **(fraction)**	**T**_**deact**_ **(s)**	**Efficacy ± SEM** **(% DA)**
Aripiprazole	WT	6.32 (6.11-6.54)	0.29 ± 0.06	243 ± 34	0.06 ± 0.01
	L94A	6.45 (6.24-6.68)	0.77 ± 0.05^****^	149 ± 11^*^	0.11 ± 0.03
Cariprazine	WT	7.28 (7.14-7.41)	0.25 ± 0.06	89 ± 7	0.06 ± 0.01
	L94A	7.32 (7.12-7.56)	0.38 ± 0.04	70 ± 7	0.13 ± 0.04
Brexpiprazole	WT	6.60 (6.35-6.85)	0.07 ± 0.02	142 ± 8	0.02 ± 0.01
	L94A	6.66 (6.55-6.77)	0.22 ± 0.05^*^	229 ± 33^*^	−0.03 ± 0.01
Lumateperone	WT	6.28 (6.07-6.48)	1.02 ± 0.13	38 ± 3	−0.04 ± 0.01
	L94A	6.84 (6.54-7.16)^**^	0.94 ± 0.03	44 ± 3	−0.04 ± 0.01

Dopamine EC_50_s of 33 nM (WT) and 41 nM (L94A) were used to calculate pK_i_s.^4^ The dopamine concentration used in the response recovery experiments was 100 μM. For ligands, 3 μM of aripiprazole, brexpiprazole, and cariprazine, and 10 μM of lumateperone were used. Concentration-response data are derived from 3 to 8 oocytes and response recovery from 4 to 7 oocytes. GIRK response recovery time constants (τ_deact_) observed during 100 μM DA application for response recovery. Monoexponential functions were fit to ~80% of the average trace responses. Efficacy was measured as currents evoked by 3 μM of aripiprazole, cariprazine, brexpiprazole, and lumateperone at D_2_R WT and L94A normalized to currents evoked by 1 μM DA in the same oocyte. F-tests (concentration-response) and t-tests (recovery and T_deact_) were used to evaluate differences between WT and L94A. ^*^, *P*<.05; ^**^, *P*<.01; ^****^, *P*<.0001

Abbreviations: D_2_R, dopamine D_2_ receptor; DA, dopamine; DPA, D_2_R partial agonist antipsychotics

The displacement of brexpiprazole and aripiprazole, having similar SPs, was similarly affected by L94A mutation, implicating the SBP. This aligns with computational and experimental results with the structural analogue SV-III-130, which shares an identical SP with aripiprazole.^4^ Cariprazine, having a PP identical to that of aripiprazole but with a distinct, carbamide-type SP, was unaffected by D_2_R L94A mutation, suggesting that the SP of cariprazine forms interactions with the SBP that are distinct from those of aripiprazole, brexpiprazole, and SV-III-130.

These results demonstrate a correspondence between SP structure and the effect of L94A mutation on induced-fit trapping of DPAs, suggesting that the SPs of these agonists interact with the SBP in the D_2_R. Thus, our findings agree better with a canonical binding mode than with an upside-down binding mode. However, we cannot rule out the potential existence of multiple binding orientations, with both canonical and upside-down binding modes coexisting and the induced-fit binding mode being canonical. It may also be possible that long-range interactions between the OBP and the SBP at the D_2_R, or between the SP and the SBP, could give rise to the observed effects even with an “upside-down” binding mode. In summary, we report evidence of induced-fit trapping of DPAs. Moreover, while there is still no experimental structure of a DPA bound to the D_2_R, our findings support a differential mode of engagement at this receptor vs 5-HT_2A_R.^5^ These data may allow for more accurate structure-based design of novel D_2_R-targeting antipsychotics.

## Data Availability

Data will be made available upon reasonable request.
